# Association of clinical predictors with recurrence of atrial fibrillation after catheter ablation

**DOI:** 10.1111/anec.12787

**Published:** 2020-07-05

**Authors:** Ang Li, Yue Chen, Wei Wang, Li Su, Zhiyu Ling

**Affiliations:** ^1^ The First Clinical College Chongqing Medical University Chongqing China; ^2^ Department of Cardiology Chongqing University Cancer Hospital Chongqing China; ^3^ Department of Cardiology Daping Hospital Third Military Medical University Chongqing China; ^4^ Department of Cardiology The Second Affiliated Hospital of Chongqing Medical University Chongqing China

**Keywords:** atrial fibrillation, catheter ablation, duration, low‐density lipoprotein cholesterol, pulmonary arterial hypertension

## Abstract

**Background:**

Recurrence rate after radiofrequency catheter ablation (RFCA) remains high, and further investigation on predictors of recurrence is needed.

**Objects:**

To identify risk factors of atrial fibrillation (AF) recurrence in patients undergoing RFCA.

**Methods:**

We retrospectively studied 257 patients with AF who underwent RFCA preceded by transthoracic echocardiographic and pulmonary vein CT examination from 2016 to 2019. Electrocardiogram examination was performed at baseline, 1, 3, 6 months, and 1 year after RFCA. We divided patients into two groups based on AF recurrence including recurrence group (*n* = 79) and nonrecurrence group (*n* = 178). The crude and independent association between clinical variables and AF recurrence was evaluated with logistic regression analysis. Specificity and positive predictive value of relevant factors for AF recurrence were performed by ROC curve.

**Results:**

Of these AF patients, 174 (68%) was paroxysmal AF and 83 (32%) was persistent AF. The multivariate logistic regression demonstrated that AF duration (OR = 1.008, 95% CI 1.002–1.013, *p* = .008), pulmonary arterial hypertension (PAH; OR = 2.313, 95% CI 1.031–5.192, *p* = .042), and low‐density lipoprotein cholesterol (LDL‐C; OR = 1.646, 95% CI 1.129–2.398, *p* = .010) were independently correlated with recurrence of AF. For predicting AF recurrence, the specificity and sensitivity of AF duration were 30.1% and 87.3%, and for LDL‐C, the specificity and sensitivity of AF duration were 60.6% and 60.5%, respectively.

**Conclusions:**

Atrial fibrillation duration, PAH, and LDL‐C might be independent risk factors for the recurrence of AF after RFCA.

## 1 INTRODUCTION

Atrial fibrillation (AF) has become the most common cardiac arrhythmia in clinical practice. The estimated morbidity of AF is between 2.3% and 3.4% in the general population and increases with aging (Ball, Carrington, McMurray, & Stewart, 2013; Lip, Brechin, & Lane, 2012). AF threatens patients’ life, increases socioeconomic and medical burden, particularly as it is associated with a fivefold increase in the risk of stroke and systemic thromboembolism and a 1.5‐ to 2‐fold increase in heart failure (Calkins et al., 2012; Fuster et al., 2011; Kirchhof et al., 2016a).

In recent years, radiofrequency catheter ablation (RFCA) has become a major effective treatment to restore sinus rhythm in patients with paroxysmal and persistent AF. However, previous evidence showed that postoperative recurrence of AF after RFCA varied from 14% to 35% in paroxysmal AF and up to 70% in persistent AF, although RFCA has been a standard therapy and continuously improved (Calkins et al., 2007; Cappato et al., 2010; Matsuo et al., 2009).Therefore, determining factors that affect AF recurrence after RFCA would be of great significance to predict the efficacy and outcomes of RFCA. It is also important for helping improve the successful rate of RFCA and guiding clinical practice.

A number of factors, mostly involved in the pathogenesis of AF, have been identified as risk factors for recurrence of AF after RFCA. The factors included advancing age, left atrial diameter, left atrial interstitial fibrosis, obstructive sleep apnea (OSAS), and serum markers such as high‐sensitive C‐reactive protein (hs‐CRP), endothelin‐1, and N‐terminal probrain natriuretic peptide (NT‐proBNP; Chun et al., 2013; Liu et al., 2011; McGann et al., 2014; Miyazaki et al., 2011; Nakazawa, Ashihara, Tsutamoto, Ito, & Horie, 2009; Staszewsky et al., 2015; Tang et al., 2009). However, discrepancy remains in different studies, and other factors associated with AF have not been thoroughly investigated. In this study, we analyzed the association between clinical variables and AF recurrence in patients post‐RFCA, and explored the factors to predict the AF recurrence.

## 2 METHODS

### 2.1 Study design and population

Patients with symptomatic AF for RFCA in the Second Affiliated Hospital of Chongqing Medical University from September 2016 to March 2019 were considered possible candidates for this study. Patients were eligible if they were admitted for their first RFCA procedure that involved circumferential pulmonary vein isolation guided by Carto Merge (Biosense Webster) electroanatomical mapping. Additionally, all subjects underwent echocardiography, 12‐lead ECG, 24‐hr Holter monitoring, and biochemical measurements before ablation. The exclusion criteria were the following: age <18 and >90 years; preoperative transesophageal echocardiography identified left atrium and/or left atrial appendage with thrombus; congenital heart disease; overt congestive heart failure; hypertrophic cardiomyopathy; and hyperthyroidism or active bleeding. The present study was performed according to the guidelines of the Declaration of Helsinki and was approved by the Ethics Committee of The Second Affiliated Hospital of Chongqing Medical University. Written informed consent was obtained from all subjects.

### 2.2 Definition of AF recurrence

Patients with paroxysmal and persistent AF were enrolled in the present study. The diagnosis of AF was confirmed by an experienced cardiologist. Paroxysmal AF was defined as terminating spontaneously or by cardioversion within 7 days of onset. Persistent AF was defined as AF lasts longer than 7 days (regardless of whether it terminates spontaneously or by cardioversion; Kirchhof et al., 2016b). Recurrence of AF was defined as any atrial tachyarrhythmia lasting for more than 30 s documented on 12‐lead ECG or 24‐hr Holter monitoring after a 3‐month blanket period of RFCA. Recurrences of AF after RFCA were generally classified into three types: (a) early recurrence (within 3 months); (b) late recurrence (from 3 months to 1 year); and (c) very late recurrence (more than 1 year). The characteristics and optimal managements differ according to those types of recurrence (Calkins et al., 2017).

### 2.3 Pulmonary pressure measurement

According to the guidelines recommendation, a diagnosis of pulmonary arterial hypertension (PAH) was confirmed by right heart catheterization at sea level and defined as the mean pulmonary artery pressure ≥25 mm Hg (Galie et al., 2016). Echocardiography was more commonly used for the measurement of pulmonary arterial pressure (PAP) because of its noninvasive nature and more routine availability. Tricuspid regurgitation pressure was measured by continuous wave Doppler combined with right atrial pressure. Pressure greater than 30 mm Hg is defined as PAH, pressure 31 to 50 mm Hg belong to mild PAH, moderate PAH at wedge 51 to 70 mm Hg, and severe PAH greater than 70 mm Hg.

### 2.4 Follow‐up

All patients were routinely followed up in the outpatient clinic at 1, 3, 6 months and later every 6 months after ablation. Physical examination, 12‐lead ECG, and 24‐hr Holter monitoring were performed simultaneously. Patients experiencing recurrence of arrhythmia symptoms were required to undergo repeat 24‐ to 72‐hr Holter monitoring.

### 2.5 Statistical analysis

All statistical analyses were performed with SPSS 19.0 software. The normally distributed variables were expressed as mean ± standard deviation (*SD*) and compared with Student *t* test, while the non‐normally distributed variables were reported as median (first and third quartiles) and compared with the Mann–Whitney *U* test; the chi‐square test was applied for comparing categorical variables. Univariate and multivariate logistic regression analysis was used to analyze the associations between clinical variables and AF recurrence. Receiver operating characteristic (ROC) curves were established to calculate areas under the curve (AUC) to evaluate the predictive values of low‐density lipoprotein cholesterol (LDL‐C) and AF for recurrence. The best cutoff values of LDL‐C and AF duration were also derived from the ROC curves. A *p* < .05 (two‐tailed) was considered statistically significant.

## 3 RESULTS

### 3.1 Subjects characteristics

A total of 257 AF patients (mean aged 60.90 ± 11.79 years; 52.9% males) were enrolled in the present study, who underwent RFCA and were followed up. Baseline characteristics of the total population and subgroups are shown in Table 1. The average follow‐up duration was 6 to 24 months. Participants were divided into two groups on the basis of AF recurrence: no‐recurrence group (*n* = 178) and recurrence group (*n* = 79). Paroxysmal AF was present in 68% of patients (70% in no‐recurrence group and 30% in recurrence group). There was no significant difference between the two groups in the baseline characteristics, including age, gender, body mass index (BMI), coronary artery disease, hypertension, diabetes mellitus (DM), chronic obstructive pulmonary disease (COPD) and previous stroke/transient ischemic attack. Patients with AF recurrence had a longer AF duration (24 vs. 36 months; *p* = .026) and higher LDL‐C level (2.21 vs. 2.53 mmol/L; *p* = .013) before ablation. As shown in Table 2, there was also no significant difference between the two groups in use of medications. As shown in Table 3, TEE analysis suggested that patients with AF recurrence had a larger left atrial diameter (LAD; 41.41 ± 8.52 vs. 44.21 ± 10.05 mm; *p* = .030) and a higher likelihood of PAH (12.9% vs. 24.1%; *p* = .026).

**TABLE 1:** Baseline characteristics of the study population (*n* = 257)

Variables	Total (257)	Nonrecurrence (*n* = 178)	Recurrence (*n* = 79)	*p* value
Age, year	60.90 ± 11.79	60.39 ± 11.66	62.06 ± 12.09	.296
Male, *n*%	156 (52.9)	109 (61.2)	47 (59.5)	.792
PAF, *n*%	174 (67.7)	122 (68.5)	52 (65.8)	.768
AF duration, months	24 (10–60)	24 (5.25–60)	36 (12–72)	.015
Smoking, *n*%	66 (25.7)	47 (26.4)	19 (24.1)	.690
BMI, kg/m^2^	24.72 ± 3.08	24.56 ± 3.08	25.07 ± 3.08	.230
Hypertension, *n*%	138 (53.7)	96 (53.9)	42 (53.2)	.909
DM, *n*%	49 (19.1)	33 (18.5）	16 (20.3)	.747
Stroke/TIA History, *n*%	26 (10.1)	18 (10.1)	8 (10.1)	.997
COPD, *n*%	10 (3.9)	7 (3.9)	3 (3.8)	.959
CHD, *n*%	60 (23.3)	38 (21.3)	22 (27.8)	.256
HDL, mmol/L	1.10 (0.93–1.28)	1.08 (0.91–1.27)	1.13 (0.93–1.33)	.125
LDL, mmol/L	2.33 (1.71–2.94)	2.21 (1.66–2.86)	2.53 (1.88–3.14)	.013
eGFR, %	88.9 ± 17.52	89.99 ± 17.26	86.53 ± 17.98	.157
Creatinine, μmol/L	73.13 ± 19.99	72.29 ± 19.95	75.09 ± 20.08	.315
UA, μmol/L	347.61 ± 102.48	341.74 ± 102.00	360.81 ± 104.19	.416
CHADS(2) score	1.48 ± 1.28	1.49 ± 1.29	1.46 ± 1.26	.804
CHA(2)DS(2)‐VASc score	2.74 ± 1.78	2.61 ± 1.82	2.78 ± 1.89	.550
Follow‐up, month	11.63 ± 3.64	11.44 ± 3.77	12.04 ± 3.35	.229

Abbreviations: BMI, body mass index; CHD, coronary atherosclerotic heart disease; COPD, chronic obstructive pulmonary diseases; DM, diabetes mellitus; HDL, high‐density lipoprotein; LDL, low‐density lipoprotein; PAF, paroxysmal atrial fibrillation; TIA, transient ischemic attack; UA, uric acid.

**TABLE 2:** Medications before and after AF ablation

Variables	Total (=257)	No recurrence(*n* = 178)	Recurrence (*n* = 79)	*p* value
Before ablation				
Amiodarone, *n*%	24 (9.3)	15 (8.4)	9 (11.4)	.451
β‐blocker, *n*%	115 (44.7)	82 (46.1)	33 (41.8)	.523
Propafenone, *n*%	8 (3.1)	6 (3.4)	2 (2.5)	.721
CCB, *n*%	32 (12.5)	23 (12.9)	9 (11.4)	.732
ACEI/ARB, *n*%	75 (29.2)	51 (28.7)	24 (30.4)	.779
Statin, *n*%	75 (29.2)	52 (29.2)	23 (29.1)	.987
After ablation				
Amiodarone, *n*%	138 (53.7)	91 (51.1)	47 (59.5)	.214
β‐blocker, *n*%	115 (44.7)	80 (44.9)	35 (44.3)	.924
Propafenone, *n*%	30 (11.7)	20 (11.2)	10 (12.7)	.743
CCB, *n*%	46 (17.9)	34 (19.1)	12 (15.2)	.450
ACEI/ARB, *n*%	119 (46.3)	84 (47.2)	35 (44.3)	.668
Statin, *n*%	182 (43.2)	126 (70.1)	56 (70.1)	.987

Abbreviations: ACEI, angiotensin‐converting enzyme inhibitor; ARB, angiotonin receptor blocker; CCB, calcium channel blocker.

**TABLE 3:** TEE findings in patients with and without atrial fibrillation recurrence

Variables	Total (*n* = 257)	No recurrence(*n* = 178)	Recurrence (*n* = 79)	*p* value
TEE				
EF, %	66.96 ± 9.60	66.68 ± 10.22	67.56 ± 8.11	.510
LVDD, mm	46.50 ± 5.84	46.56 ± 5.50	46.34 ± 6.57	.780
PAH, *n*%	42 (16.3)	23 (12.9)	19 (24.1)	.030
LAD, mm				
TD	69.07 ± 9.47	68.34 ± 8.71	70.72 ± 10.89	.090
APD	42.29 ± 9.11	41.41 ± 8.52	44.30 ± 10.11	.031
SID	55.12 ± 7.61	54.39 ± 7.32	56.80 ± 8.03	.030

Abbreviations: APD, anterior–posterior diameter; EF, ejection fraction; LAD, left atrial dimension; LVDD, left ventricular end‐diastolic dimension; PAH, pulmonary vein hypertension; SID, superior–inferior diameter; TD, transverse diameter; TEE, transesophageal echocardiography.

### 3.2 Association of clinical variables with AF recurrence

**FIGURE 1:**
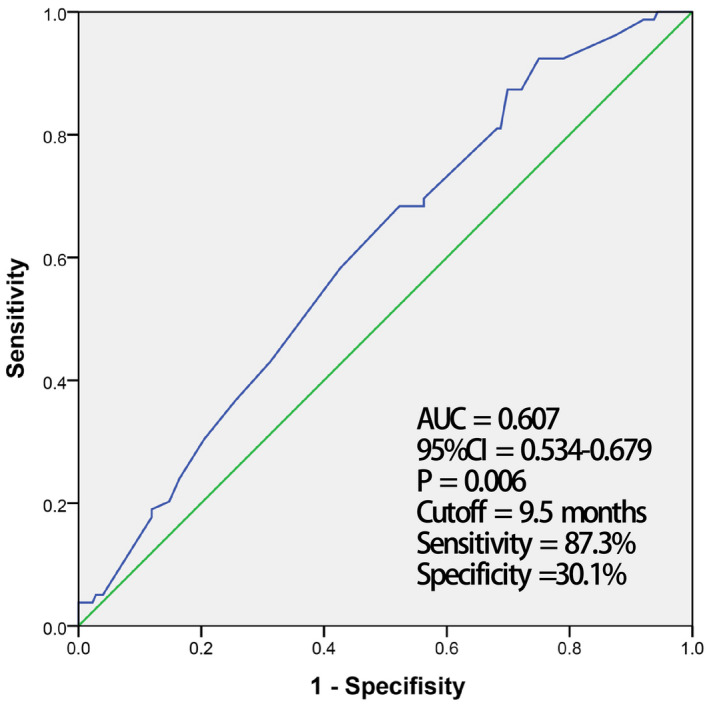
The cutoff for AF duration to predict atrial fibrillation recurrence by receiver operating characteristic (ROC) analysis

**FIGURE 2:**
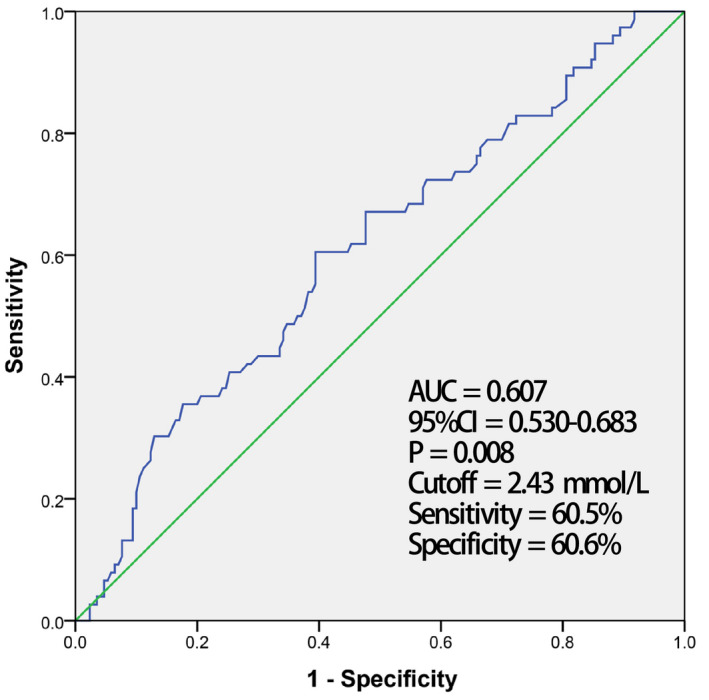
The cutoff for low‐density lipoprotein cholesterol to predict atrial fibrillation recurrence by receiver operating characteristic (ROC) analysis

The correlation between clinical variables and AF recurrence was assessed by logistic regression analysis in nonadjusted and adjusted models. Of these factors, only PAH, AF duration, and LDL‐C level were statistically significant. Patients in the recurrence group had a higher PAH prevalence (*p* = .028, OR 2.134, 95% CI 1.085–4.199), longer AF duration (*p* = .023, OR 1.005, 95% CI 1.001–1.010), and higher LDL‐C level (*p* = .014, OR 1.516, 95% CI 1.086–2.115) compared with those in the nonrecurrence group. The associations with AF recurrence remained significant following adjustment for age, gender, smoking, BMI, hypertension, DM, stroke, coronary artery disease, eGFR, statin use, high‐density lipoprotein cholesterol (HDL‐C), AF duration (*p* = .008, OR 1.008, 95% CI 1.002–1.013), and LDL‐C (*p* = .010, OR 1.646, 95% CI 1.129–2.398), PAH (*p* = .042, OR 2.313, 95% CI 1.031–5.192) separately. Additionally, we generated ROC curves to determine the 95% CI and the optimal cutoff value of AF duration and LDL‐C for the prediction of AF recurrence after RFCA. As shown in Figures 1 and 2, respectively, AF duration had an AUC of 0.607 (95% CI 0.534–0.679) with a cutoff value of 9.5 months, while LDL‐C had an AUC of 0.607 (95% CI 0.530–0.683) with a cutoff value of 2.43 mmol/L (Table 4; Figures 1 and 2).

**TABLE 4:** Predictors of AF recurrence

Characteristic	Unadjusted model		Adjusted model	
	OR (95% CI)	*p* value	OR (95% CI)	*p* value
PAH	2.134 (1.085–4.199)	.028	2.313 (1.031–5.192)	.042
AF duration	1.005 (1.001–1.010)	.023	1.008 (1.002–1.013)	.008
LDL‐C	1.516 (1.086–2.115)	.014	1.646 (1.129–2.398)	.010

Abbreviations: AF, atrial fibrillation; LDL‐C, low‐density lipoprotein cholesterol; PAH, pulmonary arterial hypertension.

## 4 DISCUSSION

In the present study, we enrolled 257 AF patients and investigated the association of clinical variables with AF recurrence after RFCA. Our data showed that patients with AF recurrence had a larger left atrial diameter, longer AF duration, more percentage of PAH, and higher level of serum LDL‐C. Logistic regression analysis identified AF duration, PAH, and LDL‐C as independent risk factors for the recurrence of AF after RFCA.

### 4.1 Pulmonary arterial hypertension

Our data showed an independent association of PAH and AF recurrence, in accordance with a previous study which enrolled 300 patients with paroxysmal AF. Among which 100 patients with PAH patients were analyzed, and it showed that PAH was independently correlated with late recurrence of paroxysmal AF after RFCA (Zhang et al., 2018). It was reported that patient with PAH revealed significantly more atrial arrhythmia incidence associated with higher mortality (Mercurio et al., 2018). A progressive increase in pulmonary vascular resistance (PVR) induces cardiac hemodynamics changes, resulting in the gradual increase in right ventricular pressure load and capacity load. The long‐standing elevation of pressure and capacity overload induces secondary right atrial and ventricular enlargement and myocardium remodeling, which may predispose the development of atrial arrhythmias (Medi et al., 2012; Simonneau et al., 2013). Conversely, atrial systolic function is gradually decreased and intraventricular pressure is increased with the onset of AF, further increasing PAP (Medi et al., 2012).

### 4.2 Atrial fibrillation duration

A positive correlation between AF duration and recurrence after ablation was observed in our study. A previous study proposed that sinus rhythm conversion rate was 75% when preoperative AF duration was <2 years, while the rate was 42% in longer‐lasting AF with left atrial dilatation (>50 mm; Grubitzsch, Grabow, Orawa, & Konertz, 2008).Several other studies have also reported on the relationship of duration of AF history prior to ablation and AF recurrence following ablation (Takigawa et al., 2014; Themistoclakis et al., 2008). It might be due to anatomic and electrical atrial remodeling with long‐term AF, but little is known about the underlying pathophysiological mechanism (Themistoclakis et al., 2008; Tokuda et al., 2011).

### 4.3 Low‐density lipoprotein cholesterol

Dyslipidemia, regarded as a major contributor to atherosclerosis and cardiovascular diseases, has been implicated as a major risk factor for ventricular arrhythmias and AF (Liu et al., 2006; Young‐Xu, et al., 2003). Particularly, it is known that high serum LDL‐C level was a risk factor for diseases including hypertension, DM, coronary artery disease, and stroke. The baseline data showed there was no significant difference of these diseases in the two groups. Still, we showed that high LDL‐C levels were positively correlated with an increased risk of AF recurrence in patients undergoing RFCA. The result was consistent with a previous study investigating serum lipid levels with AF recurrence (Aydin et al., 2014). It suggested that the lower serum LDL‐C level in the preoperative period was associated with reduced risk of AF recurrence. Meanwhile, high serum LDL‐C levels could be a predictor of AF recurrence in the postoperative period. Blood lipids can affect the composition of cell membranes and properties of cell electrophysiology. Changes of cholesterol content in the cell membrane may affect the distribution and function of ion channels, including those of the Kv1.5K^+^ channel, Kir2.1K^+^ channel, and Na^+^ channel. Those ion channel characteristics would influence Ca^2^ accumulation in the cellular membrane, which is associated with AF initiation (Bastiaanse, Hold, & Van der Laarse, 1997; Saini, Arneja, & Dhalla, 2004). Decrease in LDL‐C levels by apheresis in patients with high serum LDL‐C levels has been shown to have a protective effect against the development of AF (Aydin et al., 2014). However, whether lipid‐lowering treatment reduces the risk of AF depends on different situations. It was shown that statins reduced the postoperative risk of AF after coronary artery bypass grafting (OR, 0.52; 95% CI 0.28–0.96, *p* = .038; Marin et al., 2006). Statins have also been shown to significantly reduce the risk of AF recurrence after electrical cardioversion (RR, 0.78; 95% CI 0.67–0.90, *p* = .0003; Siu, Lau, & Tse, 2003). However, statin use after catheter ablation failed to reduce AF recurrence (Giannopoulos et al., 2018; Lee, Blaha, & Jones, 2011; Suleiman et al., 2012). Similarly, in our study, we did not find a significant association between statin therapy and AF recurrence after catheter ablation. More studies are needed to explore the relationship between lipid‐lowering treatment and AF recurrence.

### 4.4 Limitations

Our study had several limitations. First, as a cross‐sectional analysis, it could not indicate a causative relationship between increased clinical variables and reduced risk of AF recurrence. Second, because of the sample size, future studies of larger cohorts with more statistical power were needed to validate the findings of the present study. Third, some patients had a relatively short follow‐up duration (6 months), and the predictive significance of the specified factors in patients with recurrent AF warrants further evaluation. Fourth, some asymptomatic AF cases may not have been included in the study due to intermittent ECGs.

## 5 CONCLUSION

In conclusion, longer AF duration, presence of PAH, and higher LDL‐C were identified to be independent risk factors for the recurrence of AF after RFCA. Further studies of larger cohorts are required to evaluate these findings.

## CONFLICT OF INTEREST

The authors declare no potential conflicts of interest.

## AUTHORS’ CONTRIBUTIONS

Ang Li and Yue Chen concieved the study and wrote the manuscript. Ang Li, Yue chen and Li Su collected data. Statistical analysis was done by Ang Li and Yue Chen. All authors participated in discussions and interpreting data. All authors reviewed and commented on the manuscript.

## ETHICS

The study was approved by the Ethics Committee of The Second Affiliated Hospital of Chongqing Medical University.
